# Repurposed Aripiprazole-Loaded Hyaluronic Acid/Ceramide/Terpene Nanosponge as a Bioadhesive Topical Delivery System for Cutaneous *Candida albicans* Infection

**DOI:** 10.3390/ph19081135

**Published:** 2026-07-23

**Authors:** Rofida Albash, Anroop B. Nair, Mohamed A. Morsy, Katharigatta N. Venugopala, Pottathil Shinu, Mariam Hassan, Azza A. K. El-Sheikh, Amira B. Kassem

**Affiliations:** 1 Department of Pharmaceutics, College of Pharmaceutical Sciences and Drug Manufacturing, Misr University for Science and Technology (MUST), Giza 12585, Egypt; rofida.albash@must.edu.eg; 2 Department of Pharmaceutical Sciences, College of Clinical Pharmacy, King Faisal University, Al-Ahsa 31982, Saudi Arabia; momorsy@kfu.edu.sa (M.A.M.); kvenugopala@kfu.edu.sa (K.N.V.); 3 Department of Biomedical Sciences, College of Clinical Pharmacy, King Faisal University, Al-Ahsa 31982, Saudi Arabia; spottathail@kfu.edu.sa; 4 Department of Microbiology and Immunology, Faculty of Pharmacy, Cairo University, Cairo 11562, Egypt; mariam.hassan@pharma.cu.edu.eg; 5 Department of Microbiology and Immunology, Faculty of Pharmacy, Galala University, New Galala City, Suez 43511, Egypt; 6 Basic Health Sciences Department, College of Medicine, Princess Nourah bint Abdulrahman University, P.O. Box 84428, Riyadh 11671, Saudi Arabia; aaelsheikh@pnu.edu.sa; 7 Department of Clinical Pharmacy and Pharmacy Practice, Faculty of Pharmacy, Damnhour University, Damanhur 22516, Egypt; amira.kassem@pharm.dmu.edu.eg

**Keywords:** drug repurposing, aripiprazole, hyaluronic acid, ceramide, *Candida albicans*, sponge, topical drug delivery

## Abstract

**Objectives:** The present study was designed to repurpose aripiprazole (AR) as an antifungal drug for the management of topical candidiasis using a bioadhesive sponge incorporating a hyaluronic acid (HA)-, ceramide-, and terpene-based vesicular nanosystem (HCT-NS). **Methods:** AR-loaded HCT-NS were formulated by the modified ethanol injection method with varying amounts of HA, ceramide, and two types of terpenes. The formulation optimization of AR-loaded HCT-NS was carried out by a full factorial design. The responses evaluated were zeta potential (ZP), particle size (PS), and entrapment efficiency (EE). **Results:** The optimized AR-loaded HCT-NS has 5 mg of HA, 10 mg of ceramide, and fenchone, which showed spherical vesicles with EE of 81.21 ± 0.01%, PS of 223.25 ± 11.25 nm, polydispersity index (PDI) of 0.492 ± 0.003, and ZP of −27.74 ± 0.04 mV. The optimum HCT-NS showed a greater drug release in comparison to the AR suspension. In addition, the selected HCT-NS exhibited good bioadhesive characteristics and stayed stable during storage. Confocal laser scanning microscopy confirmed the penetration of the fluorescently optimized HCT-NS via the skin. Further, the scanning electron microscope image indicates the porous structure of the formed sponge. In vivo evaluations for the optimum HCT-NS sponge showed a good antifungal impact against *Candida albicans*. The safety of topical treatment was confirmed by histopathological examination. **Conclusions:** Taken together, the results here suggest that the AR-loaded HCT-NS sponge showed potent antifungal activity against *Candida albicans* fungal infection.

## 1. Introduction

Fungal infections can cause severe morbidity and mortality, ranging from minor dermatological conditions to more severe systemic infections that affect internal organs [1]. In healthcare settings, antifungal drug-resistant fungi are a particularly concerning problem because there are often no other antifungals accessible, which causes necessary therapy to be delayed or unavailable. Despite being widely used and having been crucial in the treatment of *Candida albicans* infections, many azole antifungals have become less effective due to the quick emergence of antifungal resistance [2].

Novel approaches and more all-encompassing approaches are required to enhance patient outcomes because present therapeutic alternatives are hindered by substantial difficulties that may impede the development and effectiveness of these therapies, despite the urgent need for effective antifungal medications. Finding effective and innovative antifungal medicines and/or repurposing current medications to provide new modes of action and prevent cross-resistance is urgently needed in response to these issues [3]. Furthermore, it has been determined that improving combination therapy to prevent the emergence of resistance is a strategic goal [4].

Topical drug delivery methods based on nanocarriers have received a high level of attention because of their ability to improve therapeutic effectiveness while minimizing toxicity and side effects [5]. These modern carriers improve drug retention, diffusion, and target-site concentration while lowering systemic exposure and cutaneous clearance by allowing targeted and localized drug administration within various skin layers [6]. Furthermore, nanocarriers can reduce drug doses, improve patient compliance, and extend skin residence time, making them promising platforms for effective topical and transdermal therapy [7]. The emergence of drug delivery systems utilizing nanoparticles is one potential strategy to provide innovative drug formulations for curing fungal infections and overcoming resistance to current treatments [8]. Improved medication delivery, greater solubility, and direct antifungal effects, such as the death of fungal cells and the production of reactive oxygen species, are just a few benefits of nanotechnology [9]. Despite their encouraging potential, there are still issues, such as toxicity worries, production difficulty on a large scale, and the requirement for exact control during drug release [10]. With few effective therapies, drug-resistant fungal infections pose a global health concern. Biofilm penetration, customized delivery, and efficacy are all made possible by nanotechnology [11]. It is worth mentioning that several research studies have stated the importance of utilizing nanocarriers for the drug repurposing of several drugs to effectively eradicate fungal infection. As propranolol hydrochloride was incorporated into oleosomes [12], for topical fungal infection, atorvastatin was incorporated into elastosomes for vaginal fungal infection [13], and ketoprofen was loaded into quatosomes for vaginal fungal infection [3]. Notably, aripiprazole (AR), which is well known as an antipsychotic drug, has been repurposed lately for the eradication of ocular fungal infection using terpesomes as a platform loaded in a 3D printed Ocusert [14]. On the other hand, Rajasekharan et al. confirmed that AR inhibited the development of biofilms and the production of sterols in resistant *Candida albicans* [15]. Previous studies have demonstrated that AR exhibits antifungal activity by binding to lanosterol 14-α-demethylase (CYP51/ERG11), interacting with its heme prosthetic group and inhibiting the enzyme’s catalytic activity, thereby disrupting ergosterol biosynthesis. In addition, AR suppresses HWP1 expression, resulting in inhibition of hyphal development and reduced fungal virulence [15].

On the other hand, incorporating synergistic material components within nanosystems is crucial for enhancing antifungal efficacy, as it enables complementary mechanisms of action that improve drug penetration and reduce the likelihood of resistance development. Previous studies highlighted the importance of using several additives that might enhance antifungal activity, such as terpenes [16], ceramide [17], and oleic acid [12]. On the other hand, several studies noted that the synergistic effect of nanocarriers might be related to bioadhesive and hydration properties through using materials such as hyaluronic acid (HA) [18] and pectin [19].

Nanosponges are a new formulation that encapsulates porous nanocarriers in a sponge-like shape. Because it combines the beneficial effects of microsponges and nanosized vesicular structures, it is mainly utilized for pharmacological and cosmeceutical techniques. A variety of active substances can be trapped by the porous structure, which also modifies the release pattern [20]. Previous research highlighted the importance of the efficacy of nanosponges to effectively manage topical infection, such as Fayez et al., who included voriconazole inside nanosponges [21], and Ahmed et al., who included butenafine [22].

As indicated by the current literature review, there is no scientific research that highlights the importance of using a bioadhesive formulation for AR for the topical fungal infection. Hence, our study aimed to prepare an HA/ceramide/terpene nanosystem (HCT-NS) loaded into a bioadhesive sponge to enhance the deposition of AR into the skin. A 2^3^ full factorial design was used for HCT-NS optimization to examine how the number of variables affected the physicochemical characteristics of vesicles and to choose the best HCT-NS. Entrapment efficiency (EE; Y_1_), particle size (PS; Y_2_), and zeta potential (ZP; Y_3_) were chosen as the dependent variables, whereas HA amount (mg) (X_1_), ceramide amount (mg) (X_2_), and terpene type (X_3_) were examined as independent factors. The optimum HCT-NS was assessed for its morphology, drug release, and stability. Confocal laser scanning microscopy was applied to track the penetration of the fluoro-labeled HCT-NS in skin. The optimum HCT-NS was loaded into a bioadhesive sponge for enhancing AR deposition. Finally, to assess the effectiveness and safety of the ideal HCT-NS-loaded AR system, a histological investigation and in vivo antifungal evaluation were performed in BALB/c mice.

## 2. Results and Discussion

### 2.1. Optimization of AR-Loaded HCT-NS via Full Factorial Design

The formulation of AR-loaded HCT-NS was optimized using quality-by-design methodology. By improving formulation performance, accelerating overall development, and expanding process understanding, this methodical approach fosters effective product development. The desirability function was widely used in formulation optimization to find the optimal amounts of formulation variables. Indeed, the model fitting revealed that a 2FI model was best suited to EE, PS, and ZP. All responses displayed values above 4 (Table 1), suggesting appropriate signal-to-noise ratios, confirming the model’s competence. The design space can be defined further using the model. Additionally, the design study demonstrated strong agreement between the adjusted and anticipated R^2^ values, demonstrating the model’s dependability and robustness.

#### 2.1.1. Influence on EE

An essential component used to assess the efficiency of any nanosystem is the EE. The selection of preparation technique and vesicle composition is based on the capacity of nanocarriers to entrap the drug. Analysis of variance (ANOVA) confirmed that the chosen factorial model was extremely significant (*p* < 0.0001). Excellent fit is demonstrated by the F-value (872.05) as well as a low *p*-value (*p* < 0.0001) of the model. Furthermore, model validity was reinforced by the good agreement between the adjusted R^2^ (difference < 0.2), lack of fit (*p* = 0.058), and the projected R^2^ (Table 1). The coefficient values for interactive terms (X_1_, X_2_, and X_3_) show an impact on EE, which indicates orthogonality of factors (independent factor contribution and no variance inflation), proven by variance inflation factors (VIFs are values of 1) in ANOVA data. The polynomial equation for EE% is as follows:

EE% = 76.16 − 1.87* A − 0.40* B − 4.73* C − 0.097*A* B + 0.2*A*C − 2.26 * B*C

**Figure 1:**
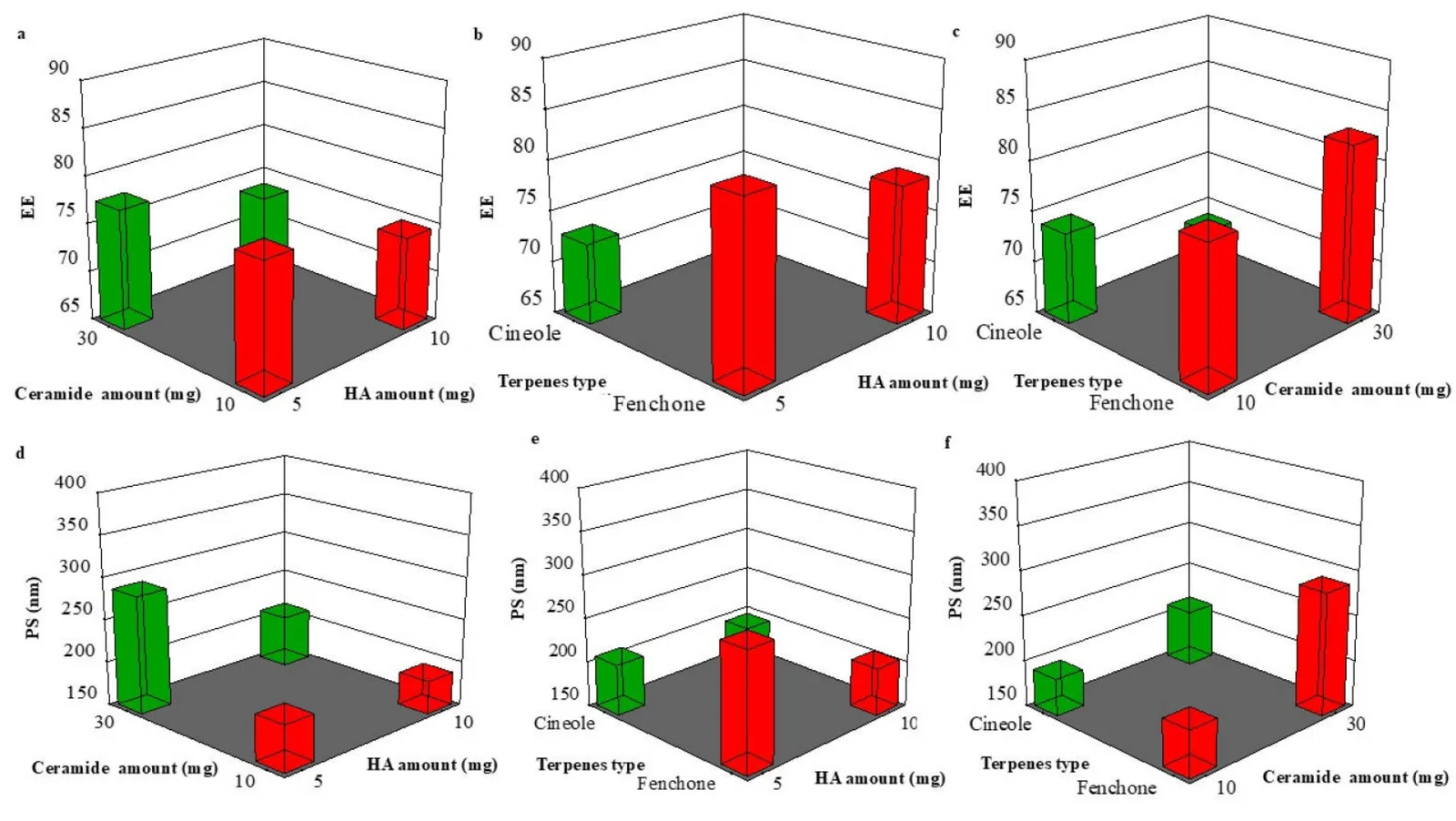
Influence of three independent variables on entrapment efficiency (EE) (**a**–**c**), and particle size (PS) of aripiprazole-loaded hyaluronic acid (HA)/ceramide/terpene nanosystem (**d**–**f**).

The EE of AR-loaded HCT-NS formulations ranged from 66.93 ± 0.03 to 84.85 ± 0.05% (Table 2 and Figure 1). These relatively high EE values are the result of the lipophilic drug’s (in this case, AR) strong binding affinity for the nonpolar areas of the ceramide and phospholipid bilayer, which facilitates its integration and keeps it inside the vesicle. For the HA amount (X_1_), the EE values decreased when the quantity of HA was increased (*p* < 0.0001). This is probably because the hydrophilic parts of HA may have contributed to the reduction in EE by preventing more hydrophobic drugs (AR) from being loaded. Our findings were in agreement with Fahmy et al. [23], who linked this decrease to interaction with minor disturbance of the bilayer membrane in vesicles, resulting in drug diffusion outside the vesicles during the preparation of elastosomes for ocular delivery of voriconazole. For the ceramide amount (X_2_) (*p* = 0.0006), it was found that EE was reduced significantly when a high quantity of ceramide (30 mg) was used. Excess ceramide can encourage lipid domain aggregation or phase separation, which further interferes with consistent drug distribution. Additionally, this could enhance drug ejection during the solidification of nanocarriers.

Regarding terpene type (X_3_), using cineole instead of fenchone resulted in lower EE (*p* < 0.0001). These observations can be explained by the lipophilic nature of the terpenes utilized, as evidenced by their log P values (log P = 2.13 and 2.82 for fenchone and cineole, respectively) [24]. Earlier studies have proved that the use of highly lipophilic terpenes generally enhances the EE of lipophilic drugs, as noticed with dapsone and finasteride [25]. It is important to mention that these results were in contrast to an earlier study wherein terpesomes of moxifloxacin hydrochloride (hydrophilic drug) were used to enhance its ocular activity [26].

#### 2.1.2. Influence on PS

Information about PS and distribution is critical for improving the effectiveness of nano-based formulations, as PS influences both drug release and absorption [27]. It is also stated that the PS and their composition can influence the bioacceptability of topical products. ANOVA confirmed that the chosen factorial model was extremely significant (*p* < 0.0001). The model shows an excellent fit with an F-value (74.57) and a low *p*-value (*p* < 0.0001). Furthermore, model validity was reinforced by the reasonable agreement between the adjusted R^2^ (difference < 0.2), the projected R^2^ (Table 1), and the lack of fit (*p* = 0.168). The coefficient values for interactive terms (X_1_, X_2_, and X_3_) show an impact on PS, which indicates orthogonality of factors, proven by variance inflation factors (VIFs are values of 1) in ANOVA data. The polynomial equation for the PS is as follows:

PS (nm) = +224.01 − 23.27* A + 27.05* B − 21.23* C − 15.75*A* B + 17.07*A*C − 16.16* B * C

Table 2 and Figure 1 indicate that the PS of AR-loaded HCT-NS formulations ranged from 180.80 ± 0.09 to 347.90 ± 7.09 nm. Indeed, the PS of the prepared AR-loaded HCT-NS formulations was significantly influenced (*p* < 0.0001) by all parameters, according to the ANOVA results. Moreover, it was also noticed that the increasing amount of HA (X_1_) (*p* < 0.0001) resulted in an increase in PS. Our findings correlated with previous research during the preparation of tamoxifen, HA-coated chitosan against breast cancer [28]. The earlier results may be connected to the binding of HA with the wall of vesicles, thus producing a coat that increases the PS as the amount of HA increases [23].

Regarding ceramide amount (X_2_), when the ceramide quantity was increased from 10 to 30 mg, it resulted in a significant increase in PS (*p* < 0.0001). This was consistent with earlier research showing that increasing the ceramide content caused aggregates to form and PS to increase. This was explained by ceramide’s inability to intercalate between membrane sheets, which could lead to its buildup on the surface of the vesicles, changing the curvature of the membrane and enlarging PS as a result [29]. Furthermore, considering the terpene type (X_3_), it significantly (*p* < 0.0001) influenced the PS, as vesicles formed by fenchone showed a larger PS than those formed by cineole. These findings could be explained by fenchone’s low lipophilicity, which creates a strong repulsive force that links the hydrophilic terpene and the lipophilic vesicular system. Consequently, because cineole is more lipophilic, terpesomes made with it had a lower PS [30].

#### 2.1.3. Influence on ZP

ANOVA confirmed that the chosen model was extremely significant (*p* < 0.0001). The model shows an excellent fit with an F-value (67.26) and a low *p*-value (*p* < 0.0001). Furthermore, model validity was reaffirmed by the reasonable agreement between the corrected R^2^ (difference < 0.2), the estimated R^2^ (shown in Table 1), and the lack of fit *p* = 0.06. Adequate precision measures the signal-to-noise ratio of 25.896, which implies sufficient signal. The coefficient values for interactive terms (X_1_, X_2_, and X_3_) show an impact on ZP, which indicates orthogonality of factors, proven by variance inflation factors (VIFs are values of 1) in ANOVA data. ZP reflects the overall surface charge of nanoparticles and serves as an indicator of nanoparticle stability. Colloidal particles with ZP values greater than ±30 mV are typically considered stable because there is enough electrostatic repulsion between particles to avoid their aggregation. The polynomial equation for ZP is as follows:

ZP (mV) = −28.21 − 0.72* A − 1.24* B − 1.74* C − 1.05*A* B − 0.24*A*C − 2.39* B* C

**Figure 2:**
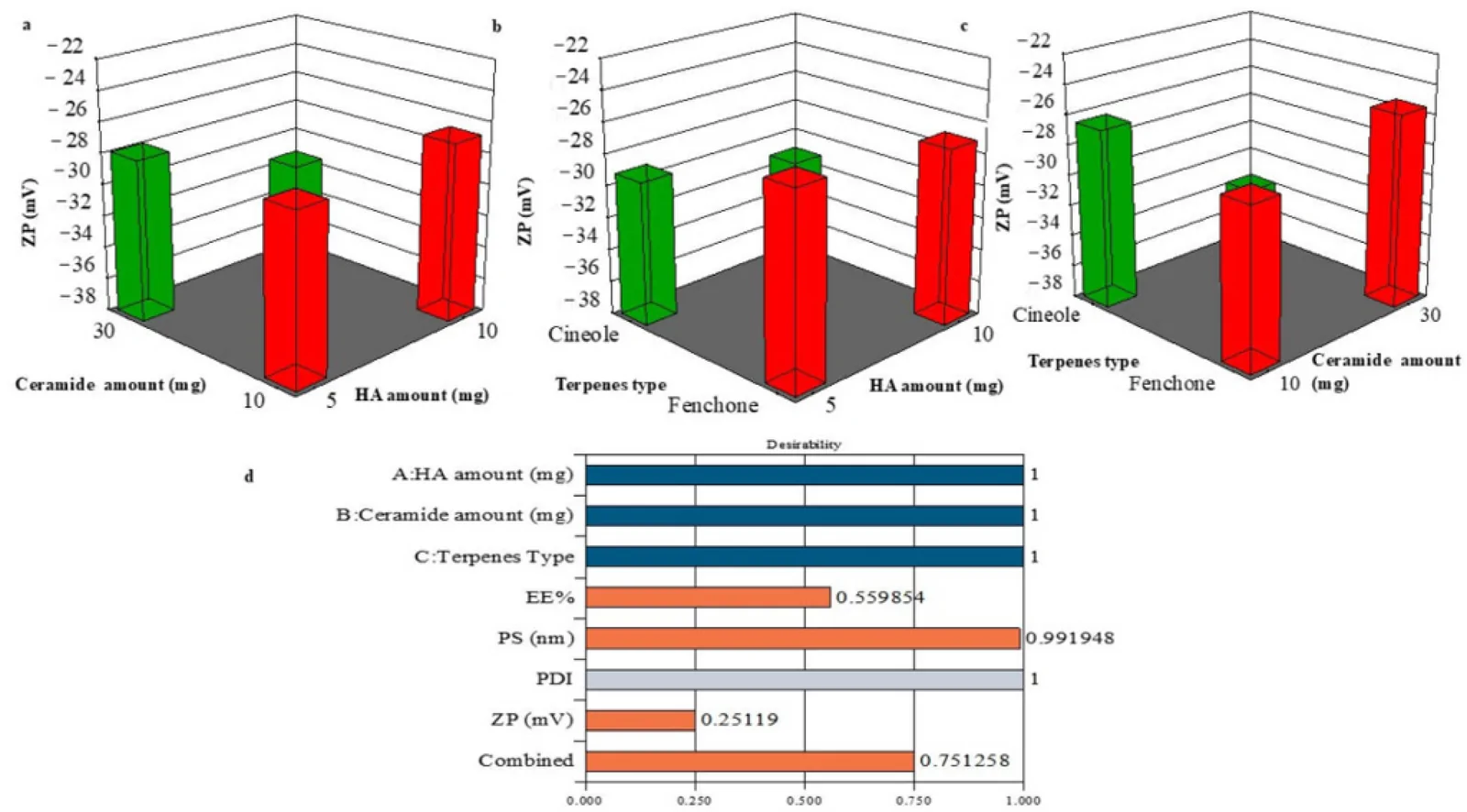
Influence of three independent variables on zeta potential (ZP) (**a**–**c**), as well as the desirability figure for optimized aripiprazole-loaded hyaluronic acid (HA)/ceramide/terpene nanosystem (**d**). EE: entrapment efficiency; PS: particle size; PDI: polydispersity index.

The ZP readings of developed AR-loaded HCT-NS formulations ranged from −24.25 ± 0.25 to −36.04 ± 0.04 (Table 2 and Figure 2) [31]. For the HA amount (X_1_), the ZP value became more negative when the HA amount became higher (*p* = 0.0024). This might be related to the presence of deprotonated carboxylate (COO^−^) groups in HA, which may increase the negative charge on the particle surface, thus contributing to the observed increase in negative ZP. Further, higher HA amounts enrich the shear plane with these anionic groups, leading to a more negative measured ZP [32].

Regarding ceramide amount (X_2_), increasing ceramide increased the negative ZP (*p* < 0.0001). The surface charge distribution may be impacted by increased lipid packing brought on by a greater ceramide amount [33]. Moreover, ceramides contain polar functional groups, particularly hydroxyl and amide groups, which can orient toward the particle surface during nanoparticle formation [34]. Taking into account the terpene type (X_3_), cineole produced more ZP than fenchone (*p* < 0.0001). This could be explained by the presence of an ether oxygen atom, which can interact with the vesicular components and affect how surface charges are distributed, improving electrostatic stabilization.

#### 2.1.4. Identification of AR-Loaded HCT-NS

Using Design-Expert^®^, which statistically examined the outcomes of all developed formulations, the best AR-loaded HCT-NS formulation was chosen. The goal of formulation optimization was to minimize PS and maximize EE and ZP (absolute value). A desirability of 0.831 was attained by the ideal formula (Figure 2). This optimum formulation was prepared using 5 mg of HA, 10 mg of ceramide, and fenchone as the terpene type, yielding an EE of 81.21 ± 0.01%, PS of 223.25 ± 11.25 nm, polydispersity index (PDI) of 0.492 ± 0.003, and ZP of −27.74 ± 0.04 mV (further supporting figures has been added as Supplementary Material for the experimental design).

### 2.2. PDI

The PS distribution range in unimodal nanoparticle populations is measured by the PDI [9]. Completely homogeneous dispersion is indicated by a PDI value of 0, whereas a highly heterogeneous, polydisperse system is indicated by a number close to 1 [35]. The PDI values of the AR-loaded HCT-NS formulations in this study varied from 0.454 ± 0.093 to 0.678 ± 0.013 (Table 2), suggesting some PS heterogeneity but remaining within the acceptable range for its measurement using dynamic light scattering [36].

### 2.3. Transmission Electron Microscopy (TEM)

**Figure 3:**
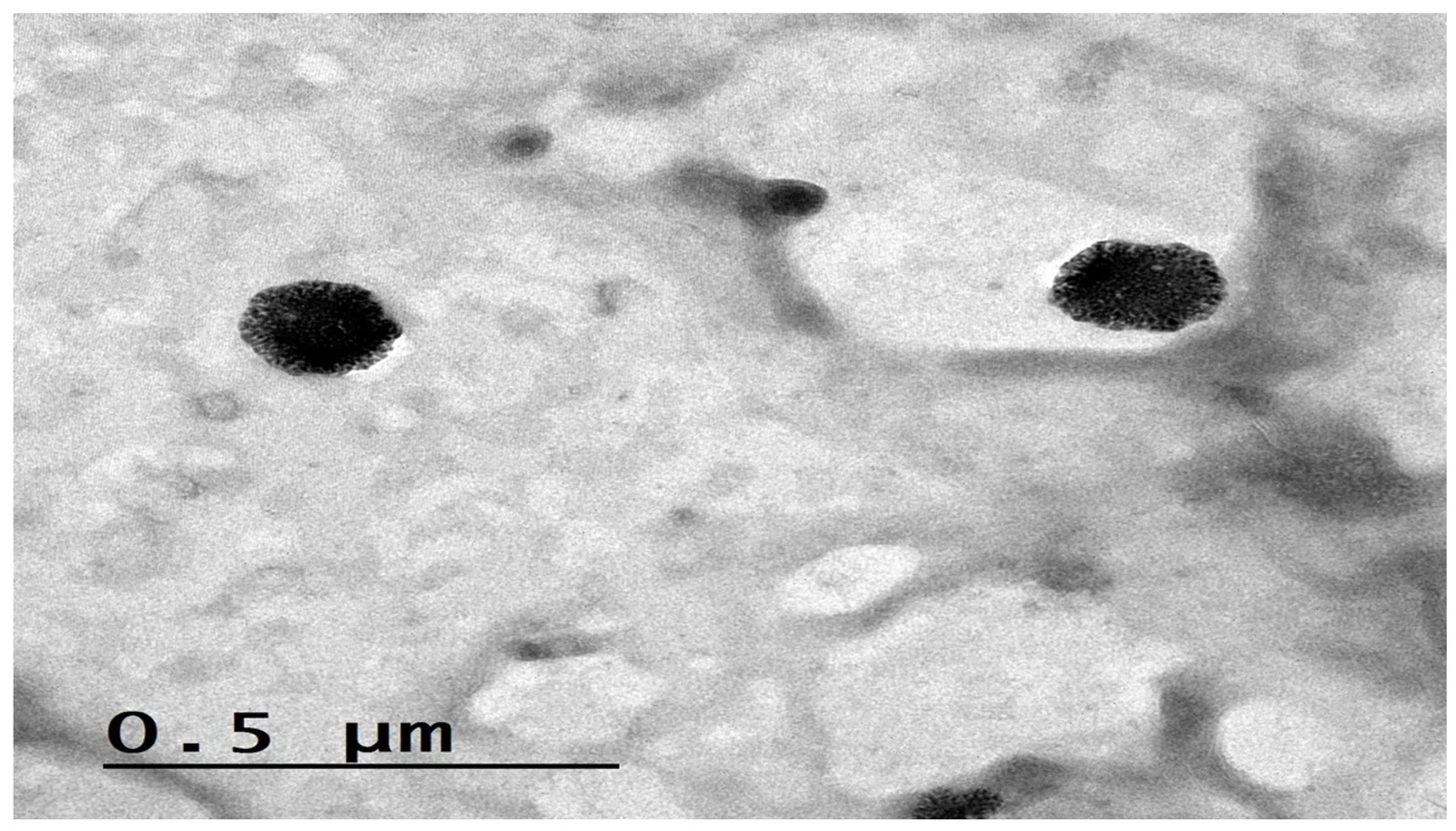
Representative transmission electron microscopy image of optimum aripiprazole-loaded hyaluronic acid/ceramide/terpene nanosystem formulation.

TEM is an imaging technique used to evaluate the morphology, shape, and size of the optimum nanoformula, hence determining its suitability for topical usage [37]. As shown in Figure 3, the AR-loaded HCT-NS formulation demonstrates a spherical shape with no aggregation or coalescence, signifying colloidal stability. The observed size is consistent with the nanometric range detected by the Zetasizer, confirming the successful formation of uniform vesicular structures. This result is also consistent with an earlier study wherein fluocinolone acetonide–hyalurosomes were developed to enhance the efficacy in rheumatoid arthritis [38].

### 2.4. Differential Scanning Calorimetry (DSC)

**Figure 4:**
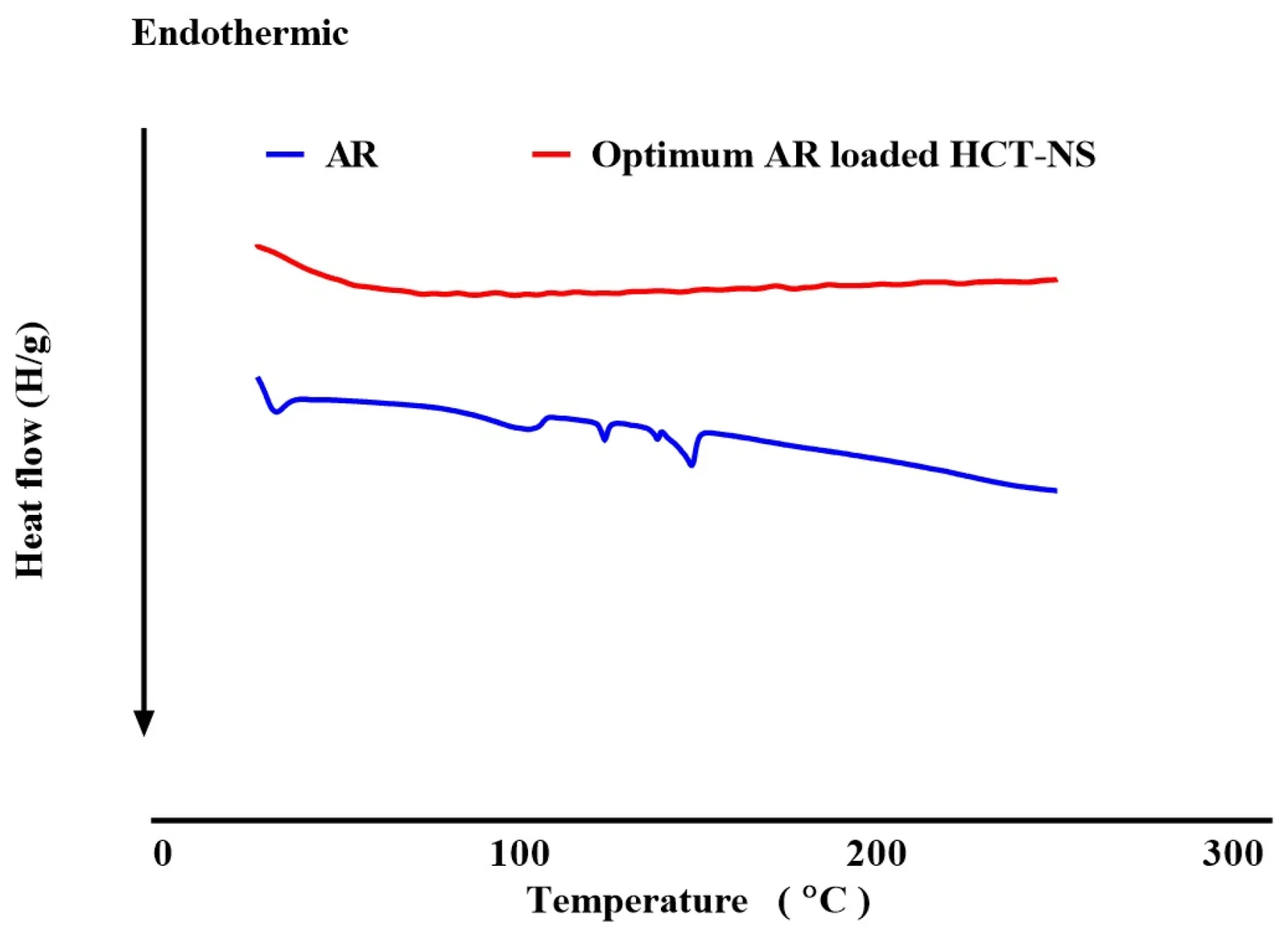
Representative thermograms of pure aripiprazole (AR) and optimum AR-loaded hyaluronic acid/ceramide/terpene nanosystem formulation.

Thermal behavior, physical state, and chemical compatibility of the drug and excipients are generally evaluated using DSC. The dehydration/melting of the hydrate form occurs below 130 °C, while the melting of the anhydrous form occurs above 130 °C, according to the thermogram of AR. The detection of a second melting point at 149 °C demonstrated the phase change to another crystal form during the heating process, although the peak at 140 °C is usually linked to the melting of the anhydrous form [39]. The thermal image of the optimized AR-loaded HCT-NS (Figure 4) revealed the absence of the AR melting endotherm (or significant broadening), indicating AR may be molecularly distributed or have changed its crystalline state to amorphous. This change is beneficial because, when compared to their crystalline counterparts, amorphous or molecularly dispersed drug forms typically show superior release properties and increased solubility. Furthermore, the absence of a distinctive AR signal confirms that AR is completely trapped within the lipid bilayer.

### 2.5. Mucin Test

A mucoadhesion measuring study was performed to assess the bioadhesive ability of the optimized AR-loaded HCT-NS on skin application [40]. Before mixing with the optimized AR-loaded HCT-NS, the mucin solution had a ZP value of −4.60 ± 1.20 mV. The ZP of optimized HCT-NS pre- and post-mixing with mucin solution were −27.74 ± 0.04 and −20.60 ± 0.86 mV, respectively. Thus, it is evident from the results that the ZP value of the mucin solution was altered to a greater negative value, which was also noticed in an earlier study [41]. This observation could be related to the negative charges on the surface of HA and ceramide. Overall, the substantial bioadhesive potential of the developed formulation was confirmed by a noticeable change in ZP readings after mixing, which was interpreted as proof of electrostatic interactions occurring between the nanovesicles and mucin chains [42]. Further, more information regarding bioadhesive performance and skin retention was considered an important aspect for future investigations.

### 2.6. Effect of Storage

The results of the storage study indicate that the physical characteristics of the optimum HCT-NS did not alter when stored for three months at low temperature (4 °C). No significant variations (*p* > 0.05) were found in EE (80.00 ± 1.20%), PS (225.00 ± 1.00 nm), PDI (0.458 ± 0.010), or ZP (−27.00.00 ± 1.00 mV) when the formulation characteristics of the kept samples were matched with the newly prepared formulations. This good stability can be because of the presence of phospholipid, which acts as a stabilizing agent to preserve vesicular membrane integrity, minimize aggregation, and maintain drug encapsulation during storage [43]. Even though the optimized formulation presented good physical stability below refrigerated conditions (4 °C), the condition for cold-chain storage may pose practical encounters for large-scale production and patient adherence. Therefore, future investigations will focus on enhancing the formulation to obtain acceptable stability under room-temperature storage conditions, assisting its clinical translation.

### 2.7. pH Evaluation

The pH of the vehicle, as well as the pH across the skin, is important for the diffusion of the drug. Previous literature stated that the optimum pH of the products for topical application on the skin should range from 4 to 7.4 [44]. The pH of the optimum HCT-NS was 5.60 ± 0.01; it is expected to be safe after topical application on the skin without causing any irritation.

### 2.8. In Vitro Release Study

**Figure 5:**
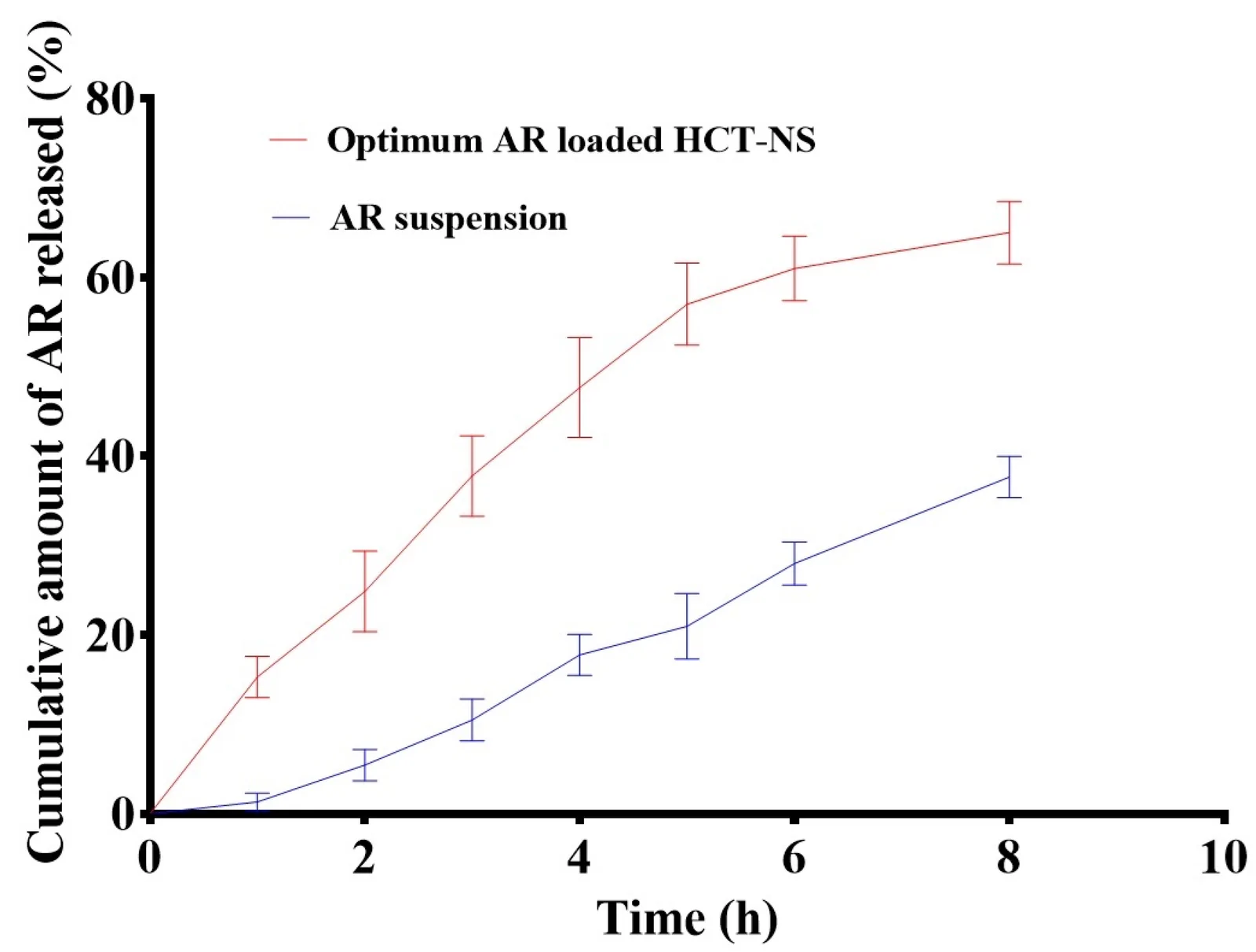
In vitro release study from aripiprazole (AR) suspension and optimum AR-loaded hyaluronic acid/ceramide/terpene nanosystem.

Drug release experiments are critical for assessing the effectiveness and therapeutic potential of topical formulations because they duplicate drug availability at the site of action. This research assures formulation uniformity, efficacy, and compatibility, as well as aids in forecasting in vivo behavior [45]. Furthermore, this test is used to assess the quality of pharmaceutical formulations [46]. The release profiles of AR suspension and optimum AR-loaded HCT-NS are presented in Figure 5. Two different release profiles were noticed, as the release of AR from the optimum AR-loaded HCT-NS is obviously greater than that from the AR suspension (Figure 5). This might be related to the capability of terpenes to solubilize AR [47]. Terpenes could increase the internal solubility of lipophilic drugs, increase porosity, and improve drug partitioning, thereby facilitating faster diffusion of the encapsulated drug, resulting in increased solubility of the entrapped low aqueous soluble drug, AR [48]. According to the calculated values of the regression coefficient, the Hixson–Crowell model (*p* = 0.965) was estimated as the best fit for AR release, which suggests that drug release is governed by changes in the surface area and particle diameter of the nanocarrier during the dissolution process. The kinetic model parameters are presented in Table 3.

### 2.9. Confocal Laser Scanning Microscopy (CLSM)

**Figure 6:**
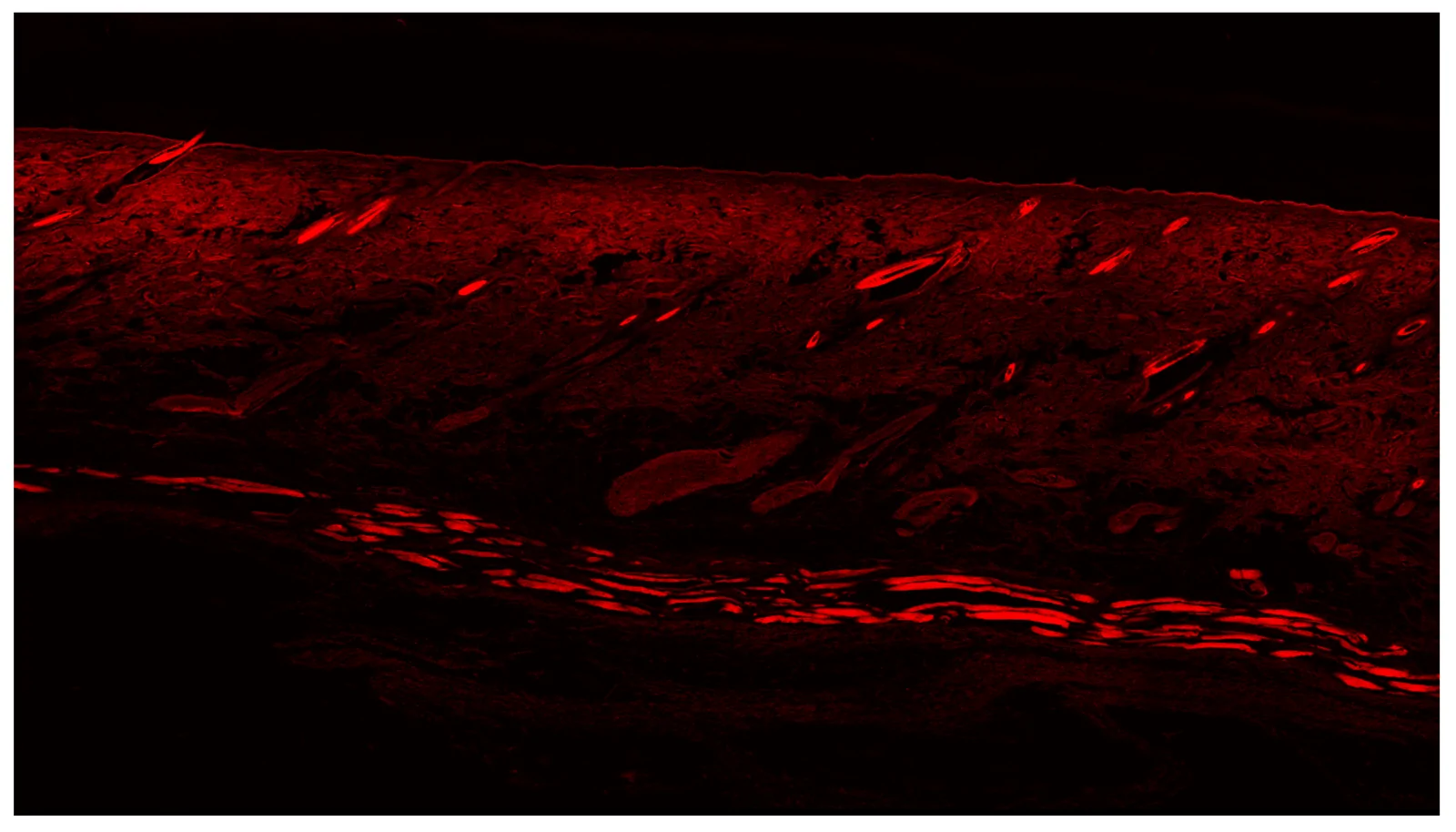
Confocal laser scanning microscopy tile images of rat skin treated with fluorescein diacetate–hyaluronic acid/ceramide/terpene nanosystem.

The infiltration efficacy and fluorescence intensity after topical application of fluorescein diacetate (FDA) labeled optimum HCT-NS were assessed by CLSM. The FDA-loaded optimal HCT-NS revealed fluorescent signals that were broadly dispersed over several skin layers, according to the CLSM image (Figure 6). The depth to which nanovesicles penetrated epidermal tissues was verified using longitudinal sections, where a consistent diffusion pattern was seen. The lower PS of the ideal HCT-NS, which improves its capacity to permeate epidermal tissues, can account for these results. These results were in agreement with those of Yang et al., who produced cerosomes to administer nicotinamide and methotrexate for the treatment of psoriasis [49].

### 2.10. Characterization of the Sponge

The formulated sponges were elegant with a smooth surface and a thickness of 1.2 ± 0.1 mm. The sponges showed an acceptable average weight variation of 76.00 ± 1.00 mg, with 0.56 ± 0.09% as the percent of friability. The in vitro disintegration time of the formulated sponges was 65 ± 12 s. The HCT-NS sponge showed significant enhancement on the release profile (59.80 ± 2.00) compared to AR suspension (37.67 ± 2.30), while it showed slightly significant sustained release compared to HCT-NS (64.99 ± 3.50) (*p* ≤ 0.05). A wash-off test was not conducted in the present research. Future investigations will include this assessment to further verify the bioadhesive aspects of the established sponge formulation.

### 2.11. Scanning Electron Microscopy (SEM)

**Figure 7:**
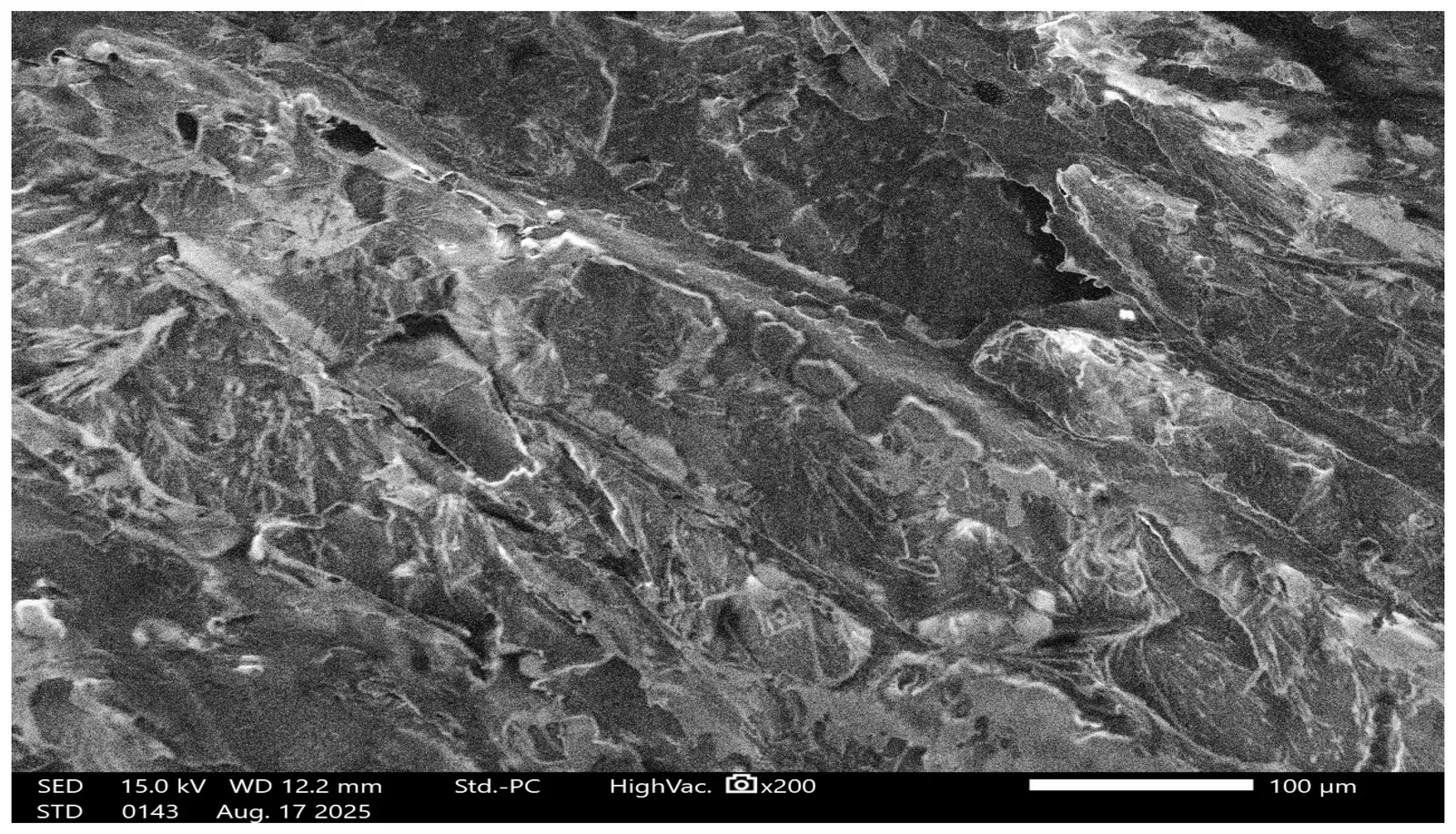
Representative scanning electron microscopy of optimum aripiprazole-loaded hyaluronic acid/ceramide/terpene nanosystem containing sponge.

SEM is frequently used to analyze the shape and PS of sponges. The porous structure of the sponge is depicted in the SEM image of optimum HCT-NS (Figure 7). The inward diffusion of HCT-NS in the polymeric solution during the production process may be the cause of the porous, spongy feature of NS that was noticed in the SEM image [22].

### 2.12. In Vivo Evaluation of the Optimum Formulation

**Figure 8:**
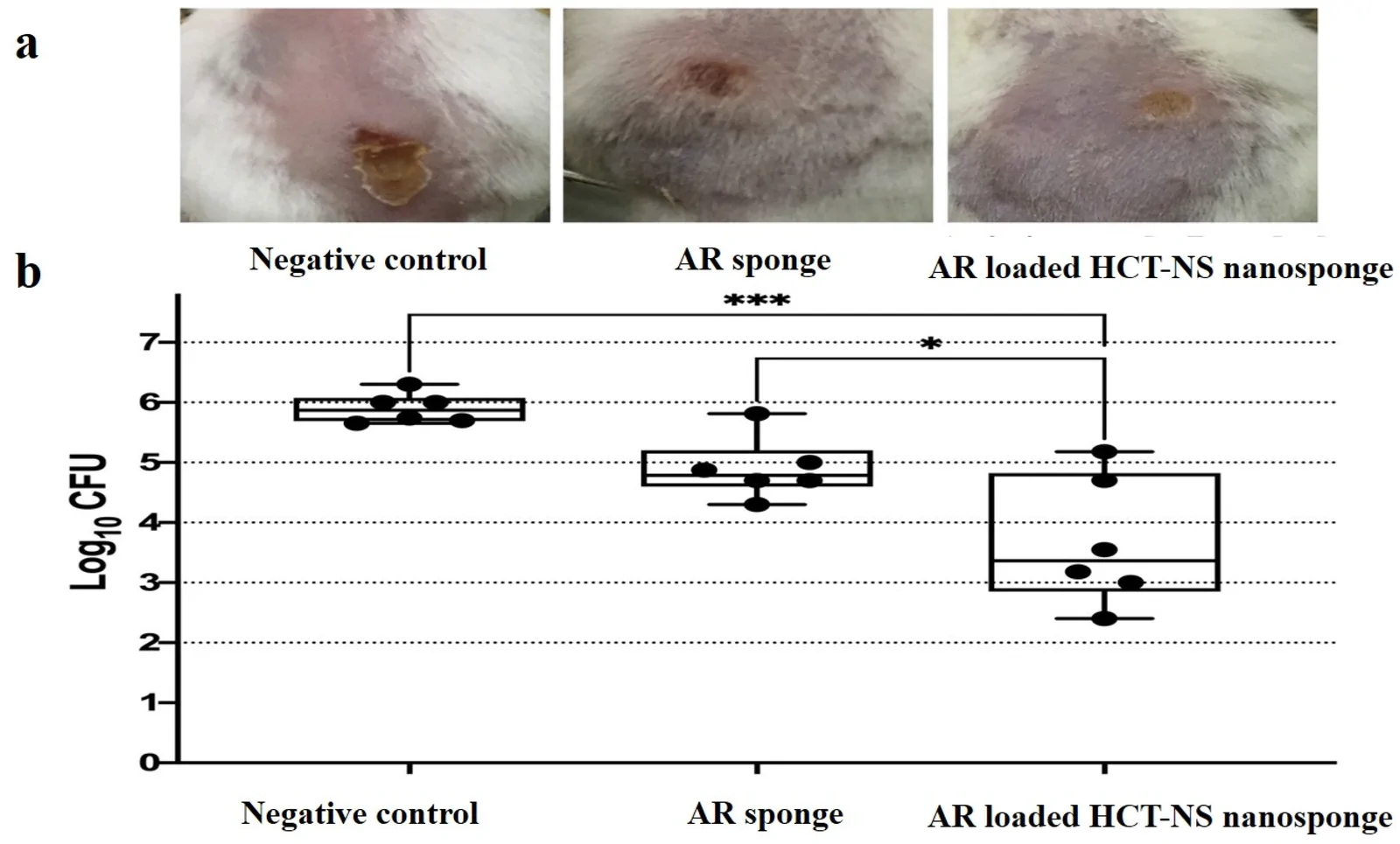
Efficacy of aripiprazole (AR) sponge and optimum AR-loaded hyaluronic acid/ceramide/terpene nanosystem containing sponge in murine model of fungal skin infection. Three groups containing eighteen BALB/c mice (n = 6) were employed. (**a**) Photographs showing the efficiency of various treated groups on fungal skin infections on the posterior backs of mice. (**b**) The effects of different treatment groups on fungal burden in a mouse model of fungal skin infection. Every point in the image represents a mouse. Values indicate the mean ± standard error. * and *** represent significance levels at *p* < 0.05 and 0.001, respectively, performed by one-way ANOVA and Tukey’s post hoc test.

The in vivo antifungal activity of AR sponge and optimum AR-loaded HCT-NS sponge was tested against *Candida albicans* ATCC 60193 using a murine skin infection model. Three groups were injected intradermally with *Candida albicans* ATCC 60193 suspension. An abscess appeared at the infection site 48 h after the infection. Visual inspection of the infected skin lesions showed a marked reduction in inflammation and crusting in the group treated with optimum AR-loaded HCT-NS sponge compared with both the negative control and the AR sponge (Figure 8a). Quantitative analysis of the fungal burden confirmed these observations (Figure 8b). The optimum AR-loaded HCT-NS sponge significantly decreased the fungal count of *Candida albicans* compared to the negative control group (one-way ANOVA, Tukey’s post hoc test, *p* < 0.001) (Figure 8). Interestingly, the AR sponge decreased the fungal count of *Candida albicans* compared to the negative control group, but was not found to be statistically significant (*p* = 0.0606) (Figure 8). Crucially, the antifungal activity of the optimum AR-loaded HCT-NS sponge was significantly superior to that of the AR sponge (one-way ANOVA, Tukey’s post hoc test, *p* = 0.02), highlighting the enhanced therapeutic performance of the nanoparticle delivery system. The fungal count recovered from the optimum AR-loaded HCT-NS sponge-treated group was 2.233 logs less than that of the negative control group. The fungal count retrieved from the AR-treated group was 1.001 logs lower than that of the negative control groups (one-way ANOVA, Tukey’s post hoc test, *p* < 0.001). The superior in vivo performance of optimum AR-loaded HCT-NS sponge compared to the free drug underscores the importance of the nano-delivery approach in repurposing non-antifungal agents [14]. Although the AR sponge showed a modest, insignificant reduction in *Candida albicans* counts, its encapsulation within the optimum HCT-NS sponge led to a significant 2.233-log reduction in fungal burden. This enhanced efficacy can likely be attributed to several factors, such as improved penetration, sustained release, and bio-adhesion. The small size of the nanoparticles may facilitate better permeation into the upper stratum corneum and infected skin layers compared to the free drug. The optimum AR-loaded HCT-NS sponge may provide a sustained release of AR at the infection site, maintaining the drug concentration above the MIC level for a longer duration [31]. The inherent bioadhesive properties of the developed sponge could improve the residence time of the formulation on the skin, preventing rapid clearance [31]. These results suggest that while AR possesses intrinsic antifungal properties, its clinical potential against cutaneous candidiasis is significantly maximized when formulated as a nanoparticle-based topical delivery system. This study’s limitation is the absence of a conventional antifungal-treated positive control group, which should be addressed in future studies.

### 2.13. Histopathological Evaluation

**Figure 9:**
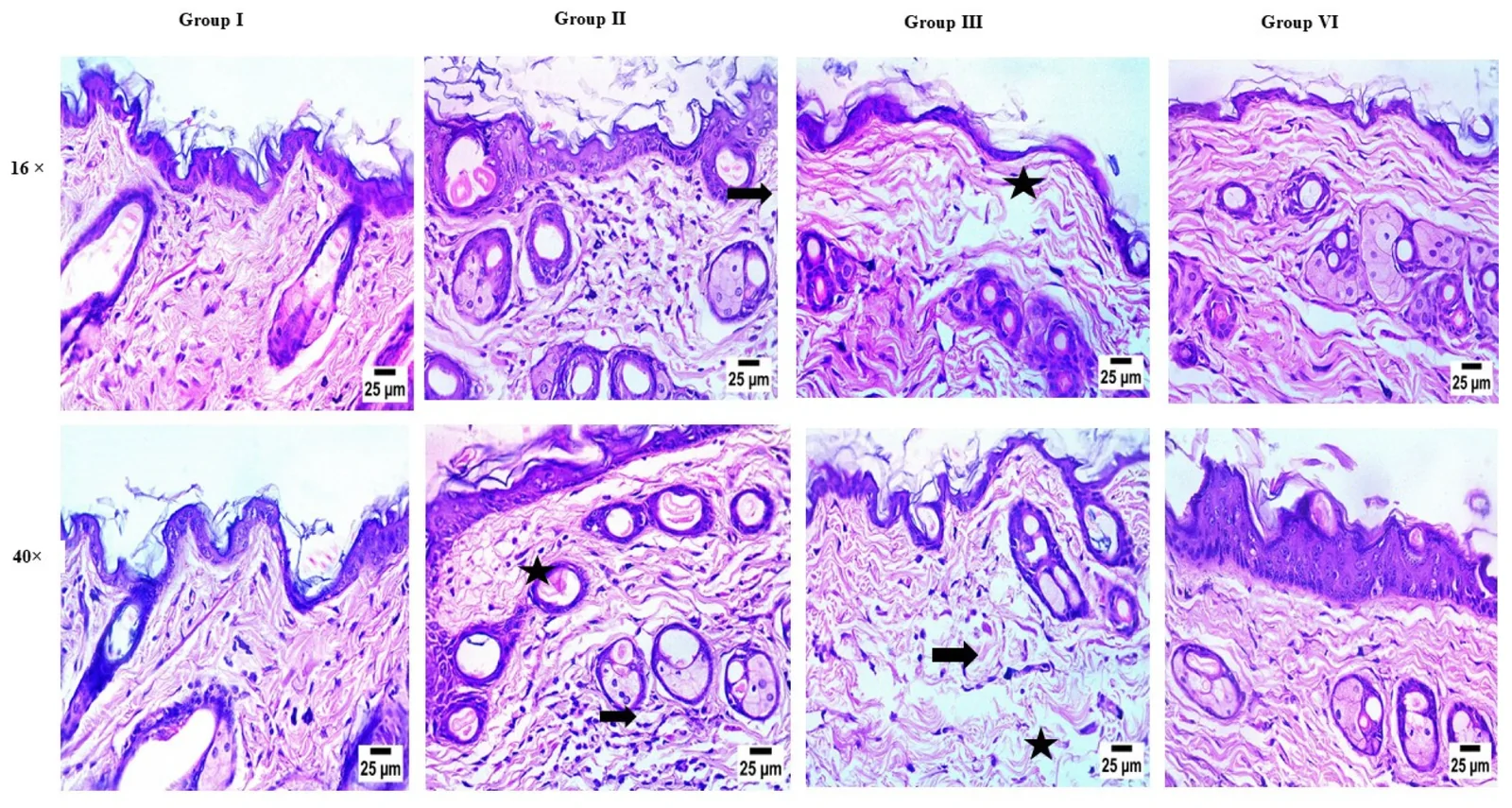
In vivo histopathological study on BALB/c mice for the negative control (Group I), positive control (Group II), aripiprazole (AR) sponge (Group III), and the optimized AR-loaded HCT-NS sponge (Group IV).

Microscopic examination (Figure 9) of rat skin samples from the normal control group (Group I) showed normal histological structures of the epidermis and dermis. Positive control (Group II) showing mild dermal edema (star) accompanied by infiltration by inflammatory cells, mainly eosinophils and lymphocytes (arrow). For the AR-treated sponge (Group III), mild dermal edema (star) was accompanied by infiltration by a few mononuclear inflammatory cells (arrow). The optimum AR-loaded HCT-NS sponge (Group IV) showed a normal histological structure of the epidermis and dermis. Overall, the histological results indicate that the optimized AR-loaded HCT-NS sponge formulation is biocompatible with skin tissue and non-irritating, successfully preserving normal skin morphology and reducing inflammatory reactions after topical administration.

## 3. Materials and Methods

### 3.1. Chemicals

The source of AR was Al Andalous Pharmaceutical Industries (Cairo, Egypt). Phospholipid (egg yolk), FDA, mannitol, glycine, pectin, Tween 80, and mucin were obtained from Sigma-Aldrich Co. (St. Louis, MO, USA). Fenchone was procured from Alfa Aesar (Karlsruhe, Germany). HA and Ceramide III were obtained from Acros Organics (Geel, Belgium) and Evonik Co. (Essen, Germany), respectively.

### 3.2. Preparation of AR-Loaded HCT-NS

AR-loaded HCT-NS was formulated by an ethanol injection method as described earlier [50]. Briefly, a mixture of ethanol/chloroform (1:1, 2 mL), phospholipid (100 mg), AR (50 mg), ceramide (III), and the terpenes (fenchone or cineole; 10 mg) was dissolved to obtain the organic phase. A 10 mL heated (60 °C) aqueous phase was slowly mixed with the organic mixture, with continuous stirring at 800 rpm using a magnetic stirrer (MSH-20D, witeg Labortechnik GmbH, Wertheim, Germany), for 30 min to remove the organic phase. The product was cooled, HA was sprinkled under continuous stirring, filtered (0.45 µm), and stored in a refrigerator.

### 3.3. Characterization of AR-Loaded HCT-NS

#### 3.3.1. EE

The free AR was isolated from the formulation (HCT-NS) by accurately weighing and centrifuging (Sigma-3K30, Sigma Laborzentrifugen GmbH, Osterode am Harz, Germany) at high rpm (20,000) for an hour at low temperature (4 °C). Additionally, the samples were washed with water (10 mL) to ensure complete removal of the AR. After that, the supernatant was diluted and quantified. A UV spectrophotometer (Shimadzu UV-1650, Kyoto, Japan) was used to measure the absorbance of samples at 255 nm [51]. A calibration curve with good linearity (y = 0.0356x + 0.0015, R^2^ = 0.99) was used to quantify the AR concentration over the concentration range of 2.5–30 μg/mL. A blank formulation with all excipients except AR was put through the same ultracentrifugation process and examined under similar conditions to verify technique specificity. At 255 nm, a negligible absorbance was observed, indicating that the formulation’s ingredients did not affect AR measurement. The calculated LOD (Limit of Detection) and LOQ (Limit of Quantification) were 1.11 and 3.35 µg, respectively.

#### 3.3.2. Physicochemical Characterization

A Zetasizer (Malvern Instruments Ltd., Worcestershire, UK) was used to measure the mean PS and PDI of HCT-NS using a dynamic light scattering procedure. Using the same device, the electrophoretic mobility of the vesicles was used to calculate the ZP. The required quantity of samples was placed in the cuvette and analyzed at room temperature. Every measurement was completed thrice [35].

### 3.4. Design for Formulation Optimization

Design-Expert^®^ (version 13, Stat-Ease Inc., Minneapolis, MN, USA) was used in a full factorial design to assess how various formulation factors influenced AR-loaded HCT-NS. The design involved eight trial runs. HA amount (mg) (X_1_), ceramide amount (mg) (X_2_), and terpene type (X_3_) were the three independent variables that were assessed (Table 4).

### 3.5. Identification of AR-Loaded HCT-NS

The optimum AR-loaded HCT-NS preparation was identified using the desirability function, which generally provides the response evaluation in experimental studies. The goal of the current study was to obtain an optimum formulation that has maximum ZP and EE while having low PS. The best formulation was determined to be the one with the highest value.

### 3.6. TEM

Using TEM (JEOL, Tokyo, Japan), the morphology and surface topography of the optimum AR-loaded HCT-NS formulation were investigated. Sample preparation was completed after diluting the formulation and placing it in a grid. The staining was completed using phosphotungstic acid (2% aqueous solution) by placing it on the grid and covering it with carbon, staining for 60 s, and allowing it to air dry [52].

### 3.7. DSC

Thermal analysis of AR and the prepared AR-loaded HCT-NS formulation was performed in a DSC-60 calorimeter (Shimadzu DSC 50; Shimadzu , Kyoto, Japan) to assess possible drug–polymer interactions and changes in crystallinity. Five milligram samples were kept in aluminum-crimped pans and sealed, with one empty pan being a reference point. Thermograms were taken as the samples were allowed to heat in the range of 10 °C to 350 °C at 5 °C/min rate. The cells were purged at 50 mL/min with dry nitrogen [53].

### 3.8. Mucin Test

The interaction of the optimum AR-loaded HCT-NS formulation with a mucin dispersion (1% *w*/*v*), a common method for forecasting skin retention, was used to evaluate the bioadhesive qualities of the mixture. To achieve homogeneous contact, equal amounts of the optimum HCT-NS and mucin solution were combined (1:1) and gently stirred with a magnetic stirrer for five minutes. To assess the level of interaction, the electrokinetic potential of both solutions (containing mucin and the optimum HCT-NS) was measured independently using a Malvern Zetasizer and compared with the electrokinetic potential of the combined system [42]. Experiments were completed in triplicate. The ZP of mucin solution alone and combined with the best formula was statistically compared by Student’s *t*-test using SPSS^®^ software 22.0. A difference at *p* ≤ 0.05 was considered significant.

### 3.9. Effect of Storage

Potential physical changes during storage were monitored in order to assess the impact of storing the optimum AR-loaded HCT-NS formulation. After three months of storage at 4 °C, different characteristics of formulations were determined and matched with the new formulations [54]. Experiments were completed in triplicate. Statistical significance was analyzed by Student’s *t*-test using SPSS^®^ software 22.0. A difference at *p* ≤ 0.05 was considered significant.

### 3.10. Skin Tolerance via pH Evaluation

To assess skin tolerance, the pH of the optimum AR-loaded HCT-NS was determined at room temperature using an Inolab pH 720 pH meter (Inolab, Weilheim, Germany). The mean of three trials ± SD was used to express the results [55].

### 3.11. In Vitro Release Study

Locally fabricated Franz cell diffusion with a diffusion area of 3.14 cm^2^ was used to measure the AR release for eight hours at 37 °C. The drug release membrane was fixed between the upper and lower chambers. Samples of optimum AR-loaded HCT-NS or AR suspension (one milliliter) were placed in the donor cell. The receiver solution consisted of 50 mL of phosphate buffer solution (pH 5.5) with 1% Tween 80 to maintain the sink condition. Samples (1 mL) were taken at different time intervals (1, 2, 3, 4, 5, 6, and 8) and analyzed in a UV spectrophotometer. Experiments were completed in triplicate. Statistical significance was analyzed by Student’s *t*-test using SPSS^®^ software 22.0. A difference at *p* ≤ 0.05 was considered significant. Further, the data from the release experiment were fitted to various kinetic models, including zero, first, second-order, Higuchi, Korsmeyer–Peppas, Hixson–Crowell, and Baker models, to determine the drug release mechanism of NIT from the TECs according to the best-fit model with the highest r^2^.

### 3.12. CLSM

This investigation was conducted to determine the penetration capacity of the prepared optimum FDA-loaded HCT-NS across various skin layers. Briefly, the formulation was prepared similarly to the method described for HCT-NS; however, FDA at 1% (*w*/*v*) was used instead of the drug. The ex vivo drug permeation was performed on a Franz diffusion setup by mounting rat skin between the donor and receptor chambers. FDA-loaded HCT-NS was applied to the dorsal side of the skin and allowed to permeate for six hours in order to simulate topical administration. Then, the skin membrane was separated from the Franz cell diffusion chamber and was cut using a rotary microtome (Model RM2245, Leica Biosystems, Wetzlar, Germany). The skin membrane was embedded in paraffin wax, and the fluorescence distribution was assessed by visualization using an inverted CLSM (Zeiss LSM710, Carl Zeiss, Oberkochen, Germany). FDA examination was completed at 497 nm (excitation) and 516 nm (emission). Skin slices were scanned using a 40× objective lens, and images were processed [56].

### 3.13. Formulation of AR Sponge and Optimum AR-Loaded HCT-NS Sponge

The lyophilization method was used to fabricate AR sponge and HCT-NS sponges [57]. Briefly, accurate amounts of HPMC K4M concentrations (1% *w*/*w*), mannitol (2% *w*/*w*), glycine (1% *w*/*w*), and pectin (2% *w*/*w*) were added to 10 mL of AR suspension or the optimum HCT-NS and were allowed to stir for 24 h to equilibrate [58]. To prepare the sponges, 1 mL of the obtained mixture was added to a circle-shaped plastic mold. To guarantee that the trapped air bubbles were removed, the loaded molds were refrigerated for 24 h. Then, the formulation was equilibrated, frozen (−25 °C) for 24 h, and allowed to lyophilize (−45 °C) for 8 h under vacuum (40 mTorr) with the help of a lyophilizer (Labconco Lyph-Lock 4.5 L Freeze Dry System, Model 77510-00, Labconco Corp., Kansas City, MO, USA).

### 3.14. Characterization of the Sponge

Weight variation, thickness, and friability of the HCT-NS-loaded sponges were evaluated to assess the uniformity and mechanical properties of the prepared formulation. Briefly, ten randomly selected sponges were individually weighed using a digital analytical balance to determine weight variation. Their thickness was measured at three different points using a digital Vernier caliper. Friability was assessed using a friabilator operated at 25 rpm for 4 min (100 revolutions), and the percentage friability was calculated from the difference between the initial and final weights of the sponges. All measurements were performed in triplicate, and the results were expressed as mean ± standard deviation [59]. The release of the formulated sponge was determined to be similar to the previously mentioned conditions for AR suspension and the optimum HCT-NS.

### 3.15. SEM

The porous nature of the optimum AR-loaded HCT-NS incorporated sponge was determined by SEM. To improve conductivity, samples were coated with gold using SEM coating equipment at room temperature in an argon environment. SEM pictures were captured at a voltage of 15 kV and a magnification of 200× [48].

### 3.16. In Vivo Study

Following the “Guide for the Care and Use of Laboratory Animals” approved by the Institute of Laboratory Animal Research (Washington, DC, USA), all animal experiments were authorized by the Research Ethics Committee of the Faculty of Pharmacy, Cairo University (Approval number MI 4108). In brief, AR was created as an HCT-NS sponge and evaluated in vivo to confirm its antifungal effectiveness in a skin infection model [54]. A local source (Cairo University animal house) provided eighteen male BALB/c mice (7 weeks old, weighing 150–200 g). They were placed in an animal house at room temperature with access to food and water. Before starting the experiment, the dorsal side of the mice was shaved. *Candida albicans* ATCC 60193 (100 µL) suspended in sterile PBS (1 × 10^9^ CFU) was injected intradermally into the dorsal area (the mice were anesthetized with ketamine [70 mg/kg i.p.]). Animals were randomly allocated to the experimental groups. Three groups were used in the current experiment, with six mice in each. Group one received topical treatment with AR-loaded HCT-NS sponge (5 mg/mL) 48 h after infection. The AR sponge (5 mg/mL) was administered to the second group. Group three was used as a negative control with no treatment. The other two groups received topical therapy at the infection site once a day for three days using the bioadhesive sponge of the designated treatment. The experiment was stopped, and the animals were killed one day after the last treatment. Then, the cutaneous lesion was removed and later homogenized with saline (0.5 mL) [60]. To measure the aerobic count, samples were diluted ten times in PBS, spread on Sabouraud dextrose agar, and incubated at 30 °C. The plates were checked for CFU after a 48-h incubation period, and the results of the test groups were compared.

### 3.17. Histopathological Examination

The animals were separated into 4 groups with 3 in each. Group I was control rats, Group II was the positive control group, and Group III was subjected to topical AR sponges, while Group IV received AR-loaded HCT-NS sponges three times daily for 1 week. After the treatment, the BALB/c mice were sacrificed, and skin specimens were collected and fixed in 10% formalin for 24 h. The tissues were fixed in paraffin blocks and sectioned with the help of a microtome (Model RM2245, Leica Biosystems, Wetzlar, Germany). The obtained sections were examined under an electric light microscope [61].

### 3.18. Data Analysis

All in vitro experiments were performed in triplicate (n = 3), and results are presented as mean ± SD. Comparisons between two groups (e.g., release, effect of storage, and mucin studies) were analyzed using Student’s *t*-test. Comparisons among multiple groups in the in vivo studies were performed using one-way ANOVA followed by Tukey’s post hoc test. Statistics were completed using SPSS^®^ software version 22.0 (SPSS^®^ Inc., Chicago, IL, USA). *p* < 0.05 was considered significant.

## 4. Conclusions

Through the formulation of AR-loaded HCT-NS, this work effectively repurposed AR, which is generally used as an antipsychotic agent, as a promising antifungal agent and improved its therapeutic effectiveness. The optimum AR-loaded HCT-NS showed high encapsulation efficiency, a nanosized range, consistent distribution, good surface charge, and greater physical stability. The developed HCT-NS offered enhanced drug release, efficient skin deposition, good bioadhesive properties, and optimum stability. Further, the fluoro-labeled optimum HCT-NS showed good deposition inside the skin by a CLSM study. The optimum AR-loaded HCT-NS was incorporated into a bioadhesive sponge to enhance drug retention and deposition. The in vivo model showed greater efficacy of the developed sponge in eradicating topical Candida infection. Further, histopathological analysis established the skin safety of the developed sponge. Overall, these findings suggest that the AR-loaded HCT-NS sponge is a promising platform for topical delivery of AR against *Candida albicans* infection. However, further preclinical and clinical studies are warranted to confirm its therapeutic potential and long-term safety.

## Figures and Tables

**Table 1:** Analysis of variance (ANOVA) table for aripiprazole-loaded hyaluronic acid/ceramide/terpene nanosystem.

Responses	R^2^	Adjusted R^2^	Predicted R^2^	Adequate Precision	Significant Factors
Entrapment efficiency, %	0.998	0.997	0.994	87.66	X_1_, X_2_, X_3_
Particle size, nm	0.983	0.967	0.937	26.55	X_1_, X_2_, X_3_
Zeta potential, mV	0.978	0.963	0.931	25.89	X_1_, X_2_, X_3_

**Table 2:** Experimental conditions and response variables for the full factorial design.

	HA Amount (mg) (X_1_)	Ceramide Amount (mg) (X_2_)	Terpenes Type (X_3_)	EE (%)	PS (nm)	PDI	ZP (mV)
F1	5	10	Fenchone	81.21 ± 0.01	223.25 ± 11.25	0.492 ± 0.003	−27.74 ± 0.04
F2	10	10	Fenchone	76.83 ± 0.01	180.80 ± 0.09	0.658 ± 0.006	−27.48 ± 0.04
F3	5	30	Fenchone	84.85 ± 0.05	347.90 ± 7.09	0.678 ± 0.013	−24.25 ± 0.25
F4	10	30	Fenchone	80.65 ± 0.15	229.00 ± 1.00	0.489 ± 0.005	−26.40 ± 0.60
F5	5	10	Cineole	75.45 ± 0.05	185.70 ± 9.70	0.524 ± 0.034	−26.85 ± 0.25
F6	10	10	Cineole	72.74 ± 0.25	198.07 ± 5.92	0.51 ± 0.005	−25.77 ± 0.01
F7	5	30	Cineole	70.59 ± 0.41	232.25 ± 0.25	0.454 ± 0.093	−31.11 ± 0.11
F8	10	30	Cineole	66.93 ± 0.03	195.07 ± 2.92	0.525 ± 0.014	−36.04 ± 0.04

HA: hyaluronic acid; EE: entrapment efficiency; PS: particle size; PDI: polydispersity index; and ZP: zeta potential.

**Table 3:** Kinetic models of release.

Model Name	Release Rate Constant (K)	Regression (R^2^)
Zero	8.445	0.926
First	−0.194	0.129
Second	0.003	0.908
Diffusion	25.544	0.964
Hixon–Crowell	0.1824	0.965
Baker	0.0149	0.963

**Table 4:** Independent variables used for aripiprazole-loaded hyaluronic acid/ceramide/terpene nanosystem optimization.

Factors (Independent Variables)	Levels	
	Low (−1)	High (+1)
X_1_: HA amount (mg)	5	10
X_2_: Ceramide amount (mg)	10	30
X_3_: Terpenes type	Fenchone	Cineole
Responses (dependent variables)	Constraints	
Y_1_: EE	Maximize	
Y_2_: PS (nm)	Minimize	
Y_3_: ZP (mV)	Maximize (absolute value)	

## Data Availability

The data presented in this study are contained within the article.

## Supplementary Materials

The following supporting information can be downloaded at: https://www.mdpi.com/article/10.3390/ph19081135/s1, Supplementary for the experimental design.
