# Supplementary material for: Repurposed Aripiprazole-Loaded Hyaluronic Acid/Ceramide/Terpene Nanosponge as a Bioadhesive Topical Delivery System for Cutaneous Candida albicans Infection
Source: Pharmaceuticals (Basel). 2026 Jul 23;19(8):1135. doi: 10.3390/ph19081135 (PMC13516225; doi:10.3390/ph19081135)
Supplement: Supplementary file 1 [file pharmaceuticals-19-01135-s001.zip › pharmaceuticals-4376256-supplementary.pdf]

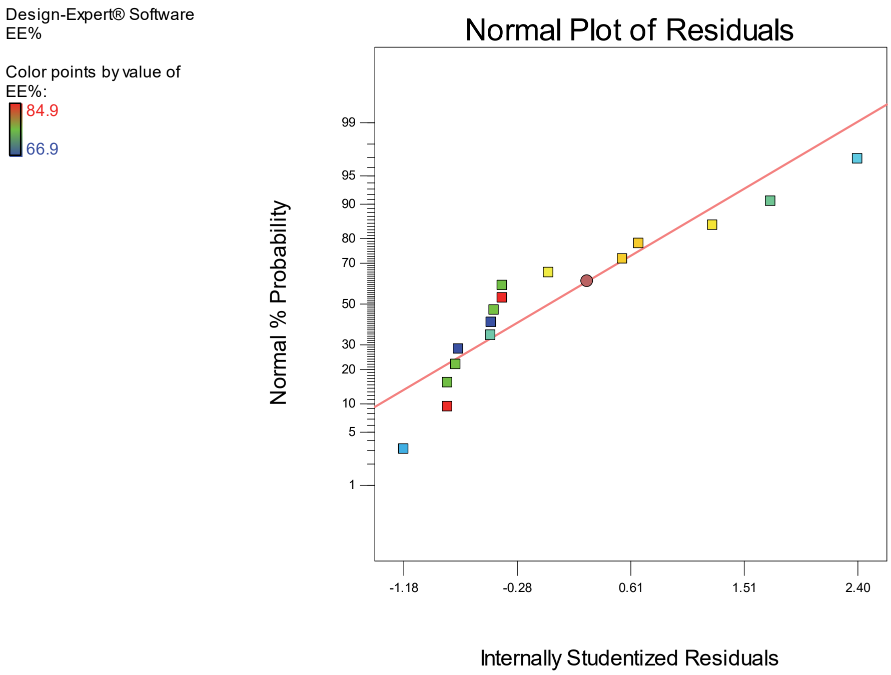
Design-Expert® Software
EE%
Color points by value of
EE%:
84.9
66.9
Normal Plot of Residuals
Normal % Probability
99
95
90
80
70
50
30
20
10
5
1
-1.18
-0.28
0.61
1.51
2.40
Internally Studentized Residuals

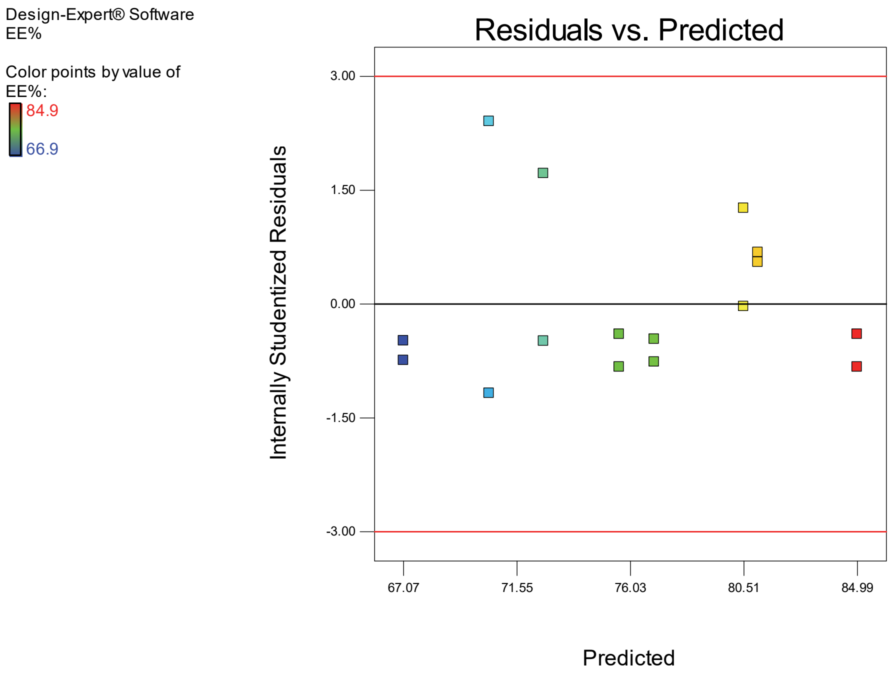

Design-Expert® Software
EE%
Color points by value of
EE%:
84.9
66.9
Residuals vs. Predicted
Internally Studentized Residuals
3.00
1.50
0.00
-1.50
-3.00
67.07
71.55
76.03
80.51
84.99
Predicted

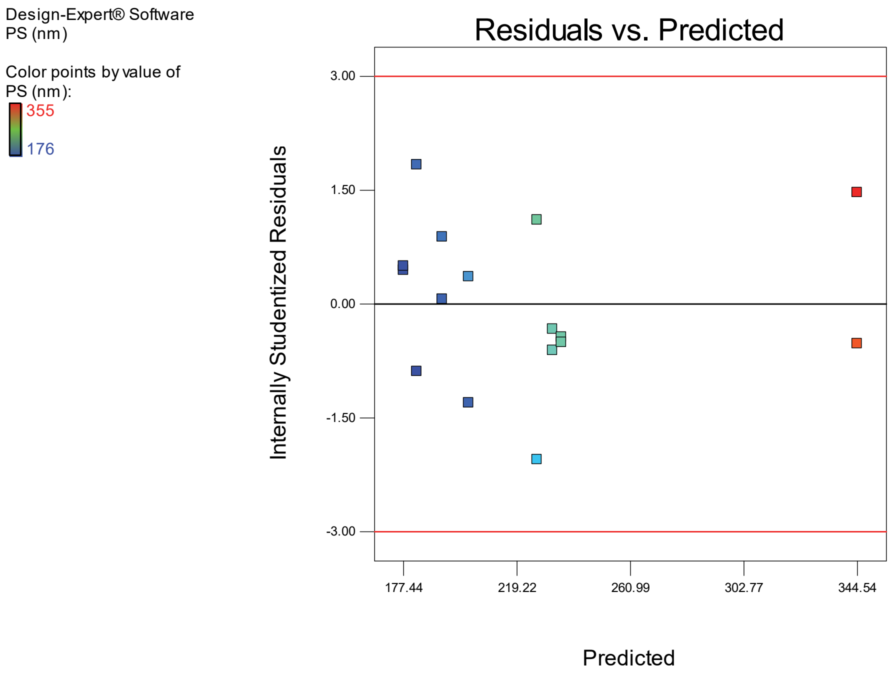
Design-Expert® Software
PS (nm)
Color points by value of
PS (nm):
355
176
Residuals vs. Predicted
Internally Studentized Residuals
3.00
1.50
0.00
-1.50
-3.00
177.44
219.22
260.99
302.77
344.54
Predicted

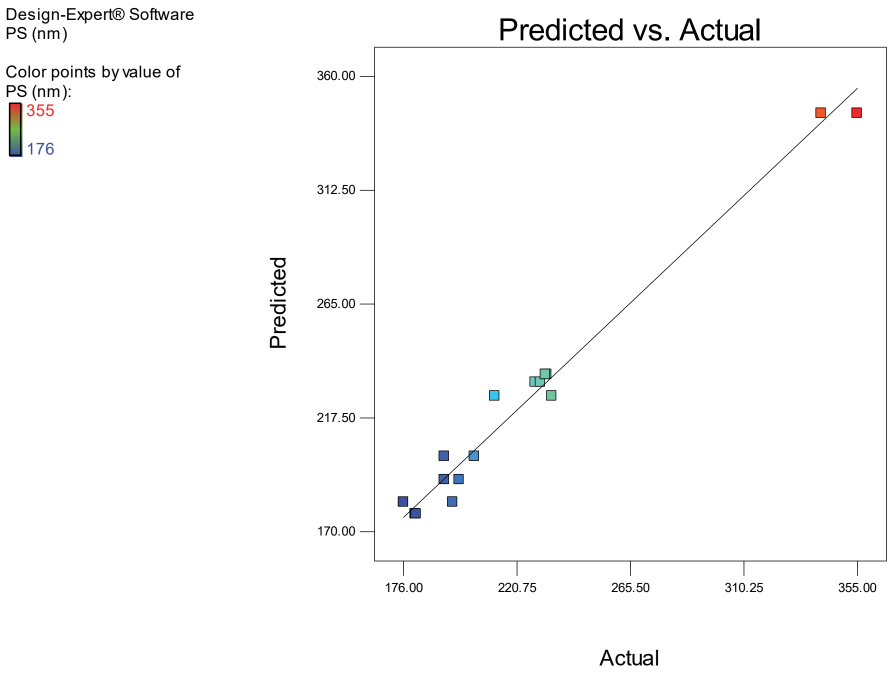
Design-Expert® Software
PS (nm)
Color points by value of
PS (nm):
355
176
Predicted vs. Actual
360.00
312.50
265.00
217.50
170.00
Predicted
176.00
220.75
265.50
310.25
355.00
Actual

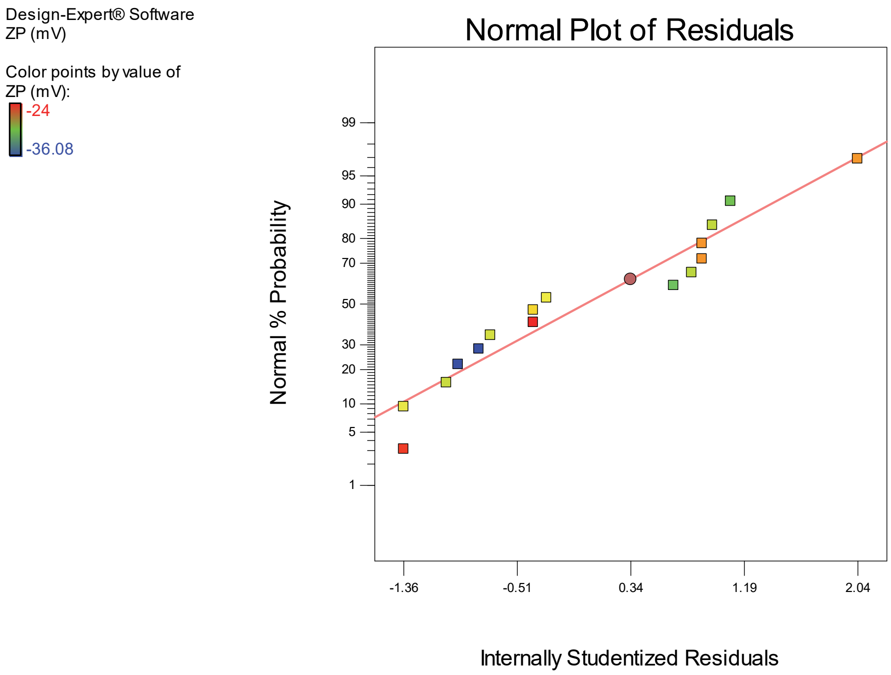
Design-Expert® Software
ZP (mV)
Color points by value of
ZP (mV):
-24
-36.08
Normal Plot of Residuals
Normal % Probability
99
95
90
80
70
50
30
20
10
5
1
-1.36
-0.51
0.34
1.19
2.04
Internally Studentized Residuals

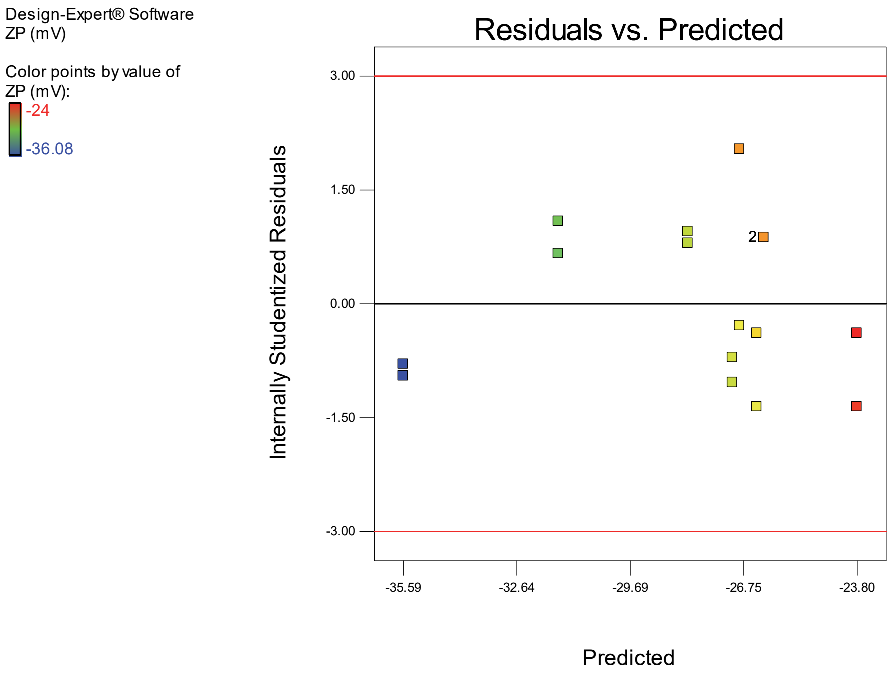
Design-Expert® Software
ZP (mV)
Color points by value of
ZP (mV):
-24
-36.08
Residuals vs. Predicted
Internally Studentized Residuals
3.00
1.50
0.00
-1.50
-3.00
2
-35.59
-32.64
-29.69
-26.75
-23.80
Predicted

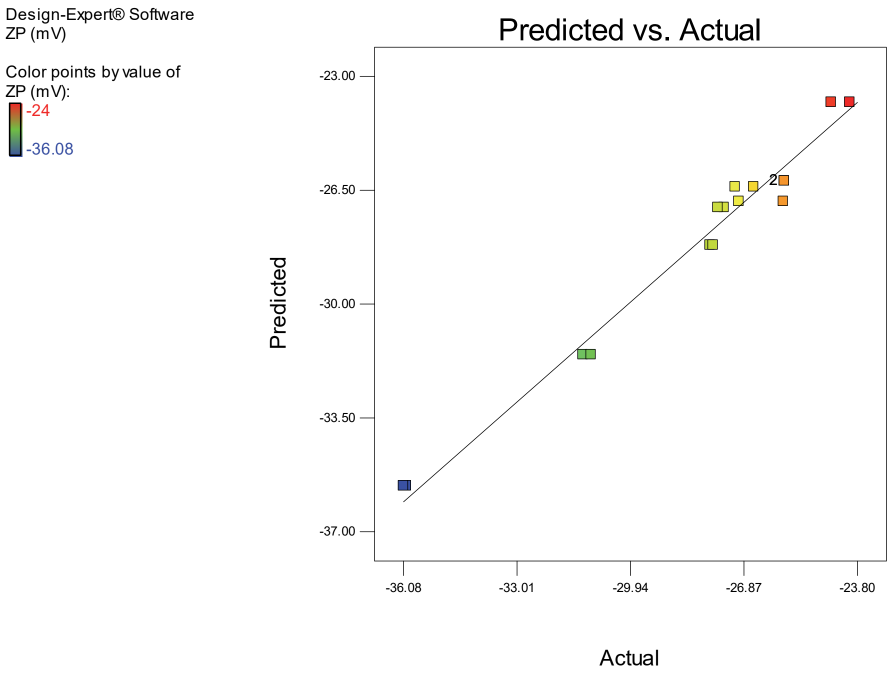

Design-Expert® Software
ZP (mV)
Color points by value of
ZP (mV):
-24
-36.08
Predicted vs. Actual
-23.00
-26.50
-30.00
-33.50
-37.00
Predicted
2
-36.08
-33.01
-29.94
-26.87
-23.80
Actual

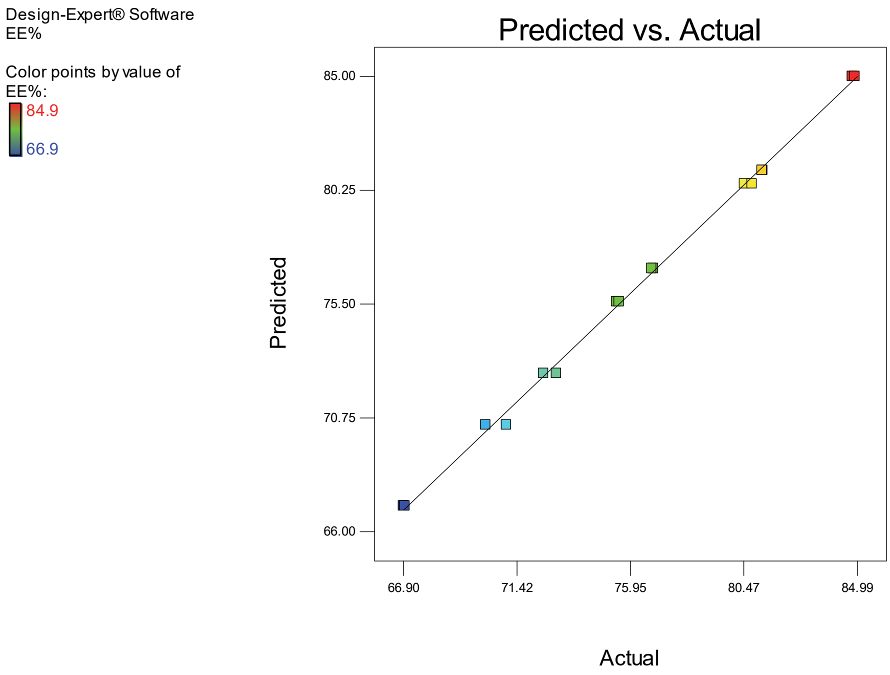

Design-Expert® Software
EE%
Color points by value of
EE%:
84.9
66.9
Predicted vs. Actual
85.00
80.25
75.50
70.75
66.00
Predicted
66.90
71.42
75.95
80.47
84.99
Actual

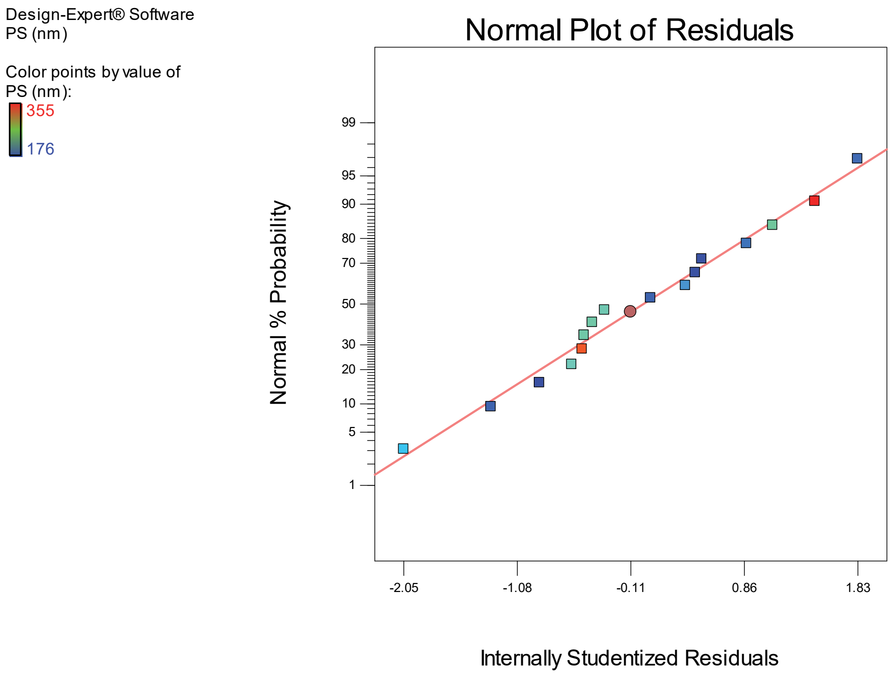

Design-Expert® Software
PS (nm)
Color points by value of
PS (nm):
355
176
Normal Plot of Residuals
Normal % Probability
99
95
90
80
70
50
30
20
10
5
1
-2.05
-1.08
-0.11
0.86
1.83
Internally Studentized Residuals
